# UBA80 and UBA52 fine-tune RNF168-dependent histone ubiquitination and DNA repair

**DOI:** 10.1016/j.jbc.2023.105043

**Published:** 2023-07-13

**Authors:** Seong-Ok Lee, Jessica L. Kelliher, Wan Song, Kyle Tengler, Aradhan Sarkar, Eloise Dray, Justin W.C. Leung

**Affiliations:** 1 Department of Pharmacology and Toxicology, College of Medicine, University of Arkansas for Medical Sciences, Little Rock, Arkansas, USA; 2 Department of Radiation Oncology, College of Medicine, University of Arkansas for Medical Sciences, Little Rock, Arkansas, USA; 3 Department of Biochemistry and Molecular Biology, College of Medicine, University of Arkansas for Medical Sciences, Little Rock, Arkansas, USA; 4 Department of Radiation Oncology, University of Texas Health Science Center at San Antonio, San Antonio, Texas, USA; 5 Department of Biochemistry and Structural Biology, University of Texas Health Science Center at San Antonio, San Antonio, Texas, USA

**Keywords:** DNA damage, nucleosome, acidic patch, 53BP1, ribosomal proteins

## Abstract

The ubiquitin signaling pathway is crucial for the DNA damage response pathway. More specifically, RNF168 is integral in regulating DNA repair proteins at damaged chromatin. However, the detailed mechanism by which RNF168 is regulated in cells is not fully understood. Here, we identify the ubiquitin-ribosomal fusion proteins UBA80 (also known as RPS27A) and UBA52 (also known as RPL40) as interacting proteins for H2A/H2AX histones and RNF168. Both UBA80 and UBA52 are recruited to laser-induced micro-irradiation DNA damage sites and are required for DNA repair. Ectopic expression of UBA80 and UBA52 inhibits RNF168-mediated H2A/H2AX ubiquitination at K13/15 and impairs 53BP1 recruitment to DNA lesions. Mechanistically, the C-terminal ribosomal fragments of UBA80 and UBA52, S27A and L40, respectively, limit RNF168-nucleosome engagement by masking the regulatory acidic residues at E143/E144 and the nucleosome acidic patch. Together, our results reveal that UBA80 and UBA52 antagonize the ubiquitination signaling pathway and fine-tune the spatiotemporal regulation of DNA repair proteins at DNA damage sites.

DNA damage occurs in cells due to exogenous and endogenous genotoxic stress (1). To maintain genome stability, our cells have evolved a coordinated DNA damage response (DDR) pathway to properly repair damaged DNA in a timely manner (2, 3). In response to DNA damage, a cohort of signaling and repair proteins are recruited to the break sites to facilitate efficient repair (4, 5). The accrual of repair proteins is primarily driven by protein–protein interaction and posttranslational modifications at the DNA breaks flanking chromatin. One of the major chromatin-based DDR pathways involves sequential signaling events of phosphorylation-ubiquitination (6, 7).

At the DNA break sites, phosphoinositide 3-kinase–related kinases, including Ataxia telangiectasia-mutated, ataxia telangiectasia and Rad3-related, and DNA-dependent protein kinase, phosphorylate histone H2AX at Serine 139 (γH2AX) (8) and initiate the ubiquitin signaling cascade *via* recruiting the MDC1–RNF8–RN168 axis to the DNA damage sites *via* protein–protein interactions (9, 10), which is at the apex of the phospho-ubiquitin DDR signaling pathway (11, 12). RNF168 catalyzes site-specific ubiquitination on the H2A family (13, 14) to generate a chromatin domain that is permissive for the ubiquitin-dependent accumulation of downstream repair proteins. More specifically, the H2A(X) K15 site-specific ubiquitination promotes self-assembly and the recruitment of downstream DNA repair proteins such as RNF169, RAD18, 53BP1, and the BARD1–BRCA1 complex at damaged chromatin to orchestrate the DDR pathway (15, 16, 17, 18, 19, 20, 21). Hence, the ubiquitination pathway is crucial in maintaining genome stability. The DDR pathway is tightly controlled to execute proper repair spatially temporally. In particular, RNF168 is involved in a key step to amplify the ubiquitin signal at damaged chromatin and a growing body of knowledge on the molecular regulation of RNF168-mediated ubiquitination at damaged chromatin (13, 22).

Recent studies showed that in addition to the catalytic RING domain, RNF168 has at least two regions that regulate target substrates specificity, namely the arginine anchor (R57, R63, R67, and R68) and the acidic region (E143/E144) resides within the UIM- and MIU-related Ubiquitin binding domain (UMI) motif. Mutations of these regions abolish the downstream 53BP1 and BRCA1 foci formation (13, 23, 24). The arginine anchor helps promotes RNF168 docking onto the nucleosome by interacting with the nucleosome acidic patch (25) while the E143/E144 residues may direct it to the target residue *via* interaction with the H2A alpha1-extension helix (13). However, if the RNF168–nucleosome interaction is constitutive, it is unclear how exactly this complex is regulated molecularly in response to DNA damage.

Here, we identify UBA80 (RPS27A) and UBA52 (RPL40), unique ubiquitin-ribosomal fusion proteins as regulatory factors for the RNF168-mediated ubiquitin signaling pathway. Through co-interaction with RNF168 and H2A/H2AX, the ribosomal fragments of UBA80 and UBA52, S27A, and L40, respectively, functionally limit the RNF168-nucleosome engagement at damaged chromatin. Depletion of UBA80 or UBA52 leads to a drastic reduction in cell proliferation, cell cycle dysregulation, and impaired DNA repair kinetics. Our findings show that the ubiquitin-ribosomal proteins precursors function as an intrinsic rheostat in regulating RNF168-mediated ubiquitin signaling and DNA repair.

## Results

### UBA80 and UBA52 bind to H2A/H2AX and RNF168

**Figure 1:**
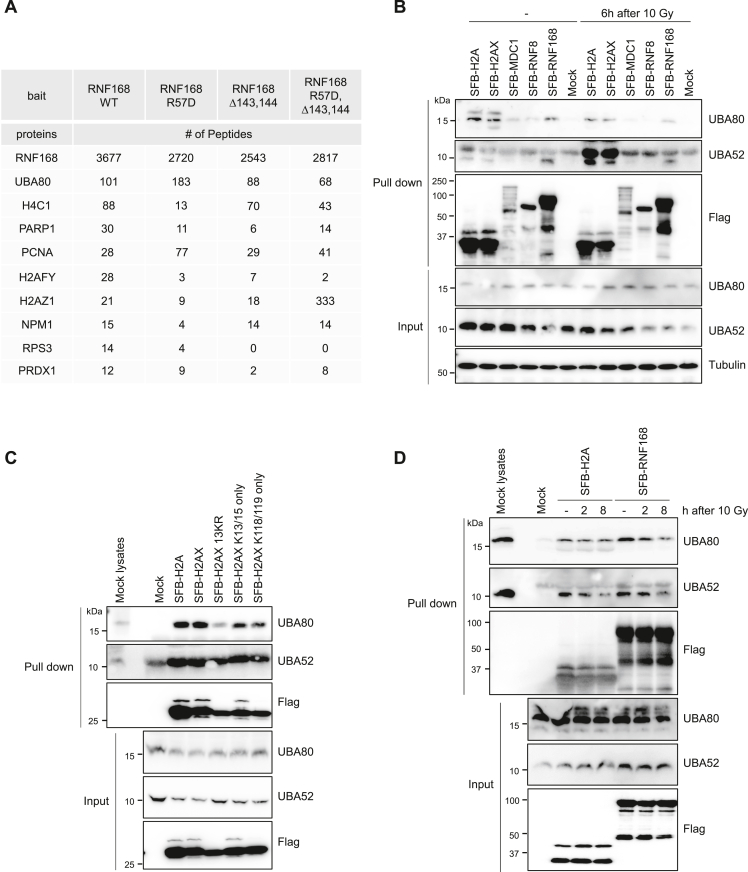
**UBA80 and UBA52 interact with H2A/H2AX and RNF168.***A,* tandem affinity purification–coupled proteomic analysis identifies UBA80 as an RNF168 interactor. The top ten list of proteins copurified with RNF168 from mass spectrometry analysis. *B,* UBA80 and UBA52 interact with both RNF168 and histone H2A. HEK293T cells were transfected with indicated SFB-tagged proteins and were irradiated with 10 Gy, allowed to recover for 6 h. Streptavidin pull-down of SFB-tagged proteins followed by Western blotting analysis as indicated. *C,* interaction between UBA80/UBA52 and H2A(X) is independent of H2A(X) ubiquitination. HEK293T cells were transfected as indicated, followed by Streptavidin pull-down and Western blotting analysis using indicated antibodies. *D,* DNA damage negatively regulates the association of UBA80/UBA52 to RNF168 and H2A(X) in cells. HEK293T cells transfected with SFB-H2A or SFB-RNF168 were irradiated at 10 Gy, allowed to recover for the indicated time, followed by Streptavidin pull-down and Western blotting analysis with specific antibodies. SFB, S-protein-FLAG–Streptavidin-binding peptide.

RNF168 has two structural entities, the arginine anchor and the acidic region (E143/E144) within the UMI domain, that regulate RNF168 target specificity (13). To identify functional partners for RNF168, we performed tandem affinity purification using S-protein-FLAG–Streptavidin-binding peptide (SFB)-tagged RNF168 followed by proteomic analysis in HEK293T cells (Fig. S1*A*). Consistent with previous reports, our data showed a number of putative RNF168–interacting proteins such as PARP1, PCNA, H2AFY (macroH2A), and H2AZ (Fig. 1*A*) (13, 26, 27). Interestingly, the ribosomal protein UBA80 was identified as the top interactor for RNF168 (Fig. 1*A*). UBA80 and UBA52 are ubiquitin fusions with the ribosomal proteins S27A and L40, respectively. Along with UBB and UBC, UBA80 and UBA52 serve as ubiquitin precursors (Fig. S1*B*). Our proteomic data identified peptides from the S27A ribosomal subunit, suggesting that the potential RNF168 interaction is not solely due to ubiquitin affinity.

To verify the proteomic data, we performed pull-down assays to determine the interaction of UBA80 and UBA52 with DNA damage response proteins. Interestingly, we observed that both UBA80 and UBA52 specifically interact with RNF168 and H2A but not with other proteins involved in the phosphorylation-ubiquitination DNA damage response axis. (Fig. 1*B*). We then performed pull-down assays using H2AX 13KR (H2AX with all 13 lysines mutated to arginine, eliminating all ubiquitination), H2AX K13/K15 only, (RNF168-mediated ubiquitination only), and H2AX K118/K119 only (RING1B/BMI1-mediated ubiquitination only) to determine whether the H2AX ubiquitination(s) are involved in their interaction. We found that UBA80 and UBA52 interact with H2AX WT and mutated forms with comparable pull-down efficiency (Fig. 1*C*). These results suggest that UBA80 and UBA52 are associated with H2A/H2AX and RNF168, independent of their ubiquitination status.

To determine if the interactions between UBA80 and UBA52 with H2A and RNF168 are DNA damage-dependent, we performed pull-down assays with SFB-H2A or SFB-RNF168 using 10 Gy of ionizing radiation (IR) and harvested the cells at the indicated time points. Both UBA80 and UBA52 showed a reduction in RNF168 interaction after IR. UBA52 showed a more noticeable reduction in H2A binding compared to UBA80 (Fig. 1*D*). Collectively, these data suggest that UBA80 and UBA52 bind to both RNF168 and H2A, and their interactions are negatively regulated following DNA damage.

### UBA80 and UBA52 are recruited to DNA damage sites

**Figure 2:**
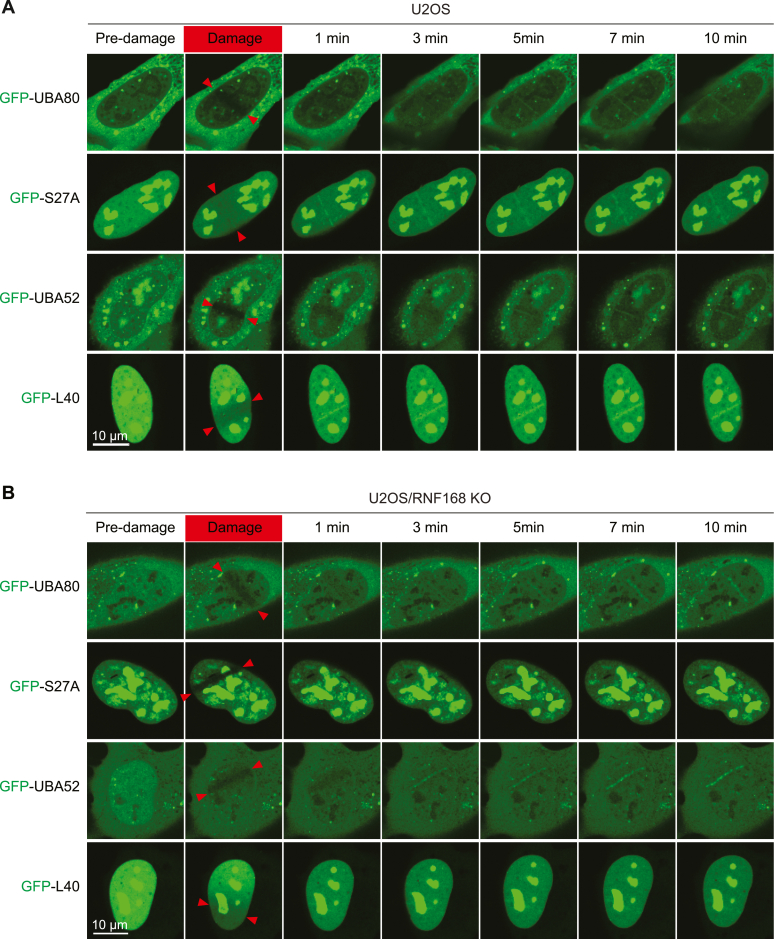
**UBA80 and UBA52 are recruited to DNA damage sites.***A* and *B,* U2OS cells (*A*) and U2OS/RNF168 KO cells (*B*) with GFP-protein expression were treated with laser-induced micro-irradiation and analyzed at indicated time points by confocal microscopy. *Red arrows* indicate the laser path.

UBA80 and UBA52, as ubiquitin-ribosomal fusion proteins, are posttranslationally processed (28, 29, 30). Consistent with previous reports, N-terminal GFP-tagged UBA80 and UBA52 showed ubiquitin conjugation patterns similar to GFP-Ub (Fig. S2, *A*–*C*). Conversely, UBA80 and UBA52 cleavage-resistant mutants did not show any ubiquitin-conjugation pattern (Fig. S2, *A*–*C*) (28). For C-terminal GFP-tagged UBA80 and UBA52, we detected a major band with the same size as the N-terminal GFP-tagged S27A and L40, respectively. Furthermore, we observed that GFP-UBA80 or GFP-UBA52 have different subcellular localization compared to GFP-S27A or GFP-L40. While GFP-UBA80 and GFP-UBA52 localize in both cytosol and nucleoli, GFP-S27A and GFP-L40 localize in the nucleus and primarily in the nucleoli where the ribosome assembly occurs (Fig. 2, *A* and *B*). Together, these data support the notion that these precursors are processed and cleaved posttranslationally into separate ubiquitin and ribosomal proteins.

Next, we assessed the ability of the UBA80 and UBA52 cleaved products to be recruited to sites of DNA damage. We observed the recruitment of GFP-UBA80 and GFP-UBA52 5 min after damage at the laser micro-irradiation–induced DNA breaks. The recruitments likely represent ubiquitin conjugation, at DNA damage (Fig. 2*A*). Notably, the ribosomal S27A and L40 fragments were also accumulated at DNA damage sites at an earlier time point (Fig. 2*A*) and their recruitments are RNF168 dependent (Fig. 2*B*), suggesting they may also play a regulatory role in the DDR pathway in collaboration with RNF168. Interestingly, UBA80 and UBA52 cleavage-resistant mutants were not recruited to micro-irradiation–induced damage sites (Fig. S2*D*), suggesting that the posttranslation cleavage of the UBA proteins is required for their damage chromatin localization.

A previous report has shown that UBA80 is mono-ubiquitinated at lysine 113 (31). Despite the interactions, RNF168 did not show ubiquitination activity to UBA80 or UBA52 (Fig. S2*E*). With RNF168 overexpression, we observed increased ubiquitination of H2AX, but not endogenous or GFP-tagged S27A and L40, indicating RNF168 is not an E3 enzyme for UBA80 ubiquitination (Fig. S2*E*). Moreover, UBA80 and UBA52 ubiquitination does not seem to be induced by DNA damage (Fig. S2*F*).

### UBA80 and UBA52 depletion impair DNA repair kinetics

**Figure 3:**
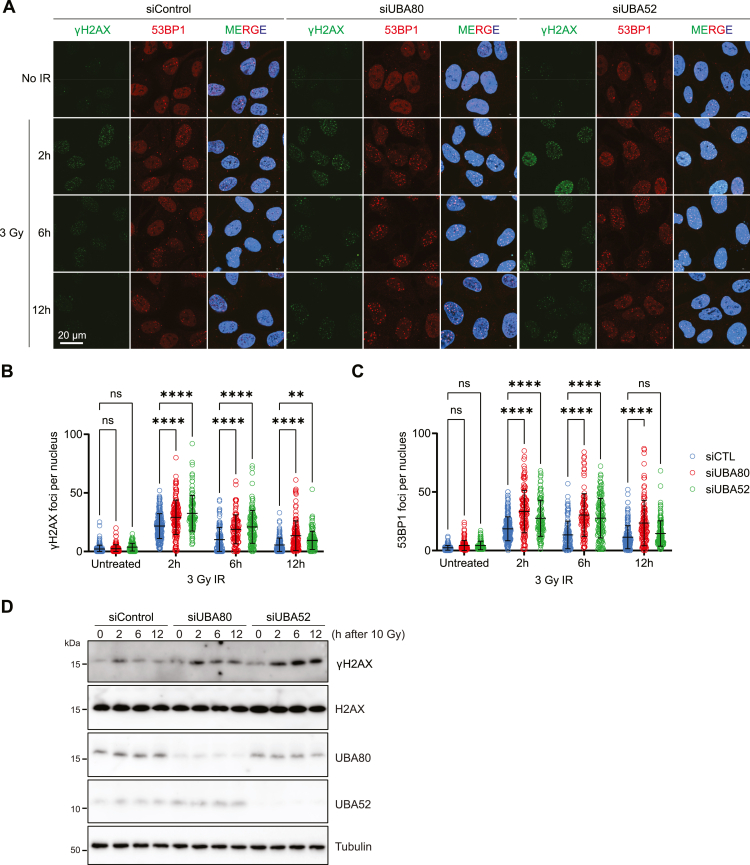
**UBA80 and UBA52 are required for proper DNA damage response.***A,* UBA80 and UBA52 depletion impair DNA repair kinetics. U2OS cells were transfected with indicated siRNAs. After 72 h, cells were irradiated with 3 Gy or untreated and fixed as indicated time, then followed by immunofluorescence analysis with γH2AX and 53BP1 antibodies. *B* and *C,* quantification of nuclear γH2AX and 53BP1 foci in *A*, each *dot* represents a single cell. For each condition, images containing at least 100 cells were acquired. Data presented as mean±SD. Two-way ANOVA was used for statistical analysis. *∗∗∗∗p < 0.0001. D,* UBA80 and UBA52 depletion leads to persistent DNA breaks. U2OS cells were transfected with indicated siRNA. After 72 h, cells were irradiated with 10 Gy and harvested at the indicated time point in a 1× Laemmli sample buffer, followed by Western blot analysis with indicated antibodies.

We then use siRNA-mediated knockdown to deplete UBA80 and UBA52 in cells and investigate their function in DNA repair. Depletion of UBA80 or UBA52 did not alter the protein level of ubiquitin, H2AX, RNF8, and RNF168 (Fig. S3*A*). Strikingly, UBA80- and UBA52-depleted cells showed increased γH2AX and 53BP1 IR induced–foci formation (IRIF) at 2, 6, and 12 h time points after IR without a significant difference in undamaged cells (Fig. 3, *A*–*C*), suggesting that DNA breaks persist, and the repair kinetics are slower in the absence of UBA80 and UBA52. Similarly, immunofluorescence quantification showed that γH2AX and MDC1 levels are higher in UBA80- and UBA52-depleted cells (Figs. 3*D* and S3, *B*–*D*) indicative of persistent DNA damage (32).

Surprisingly, depletion of UBA80 or UBA52 reduces BRCA1 IRIF, to a greater extent in the UBA80-depleted cells (Fig. S3, *C* and *D*). Consistent with a previous report (33), cell cycle analysis revealed that UBA80 depletion leads to G1 arrest in the cell cycle, which potentially contributes to the drastic reduction of BRCA1 IRIF (Fig. S3*E*). On the contrary, UBA52-depleted cells displayed an arrest in the S/G2 phase (Fig. S3*E*). Consistent with the cell cycle dysregulation, UBA80 and UBA52 depletion leads to proliferation defects in cells (Fig. S3*F*).

### UBA80 and UBA52 inhibit 53BP1 foci formation upon DNA damage

**Figure 4:**
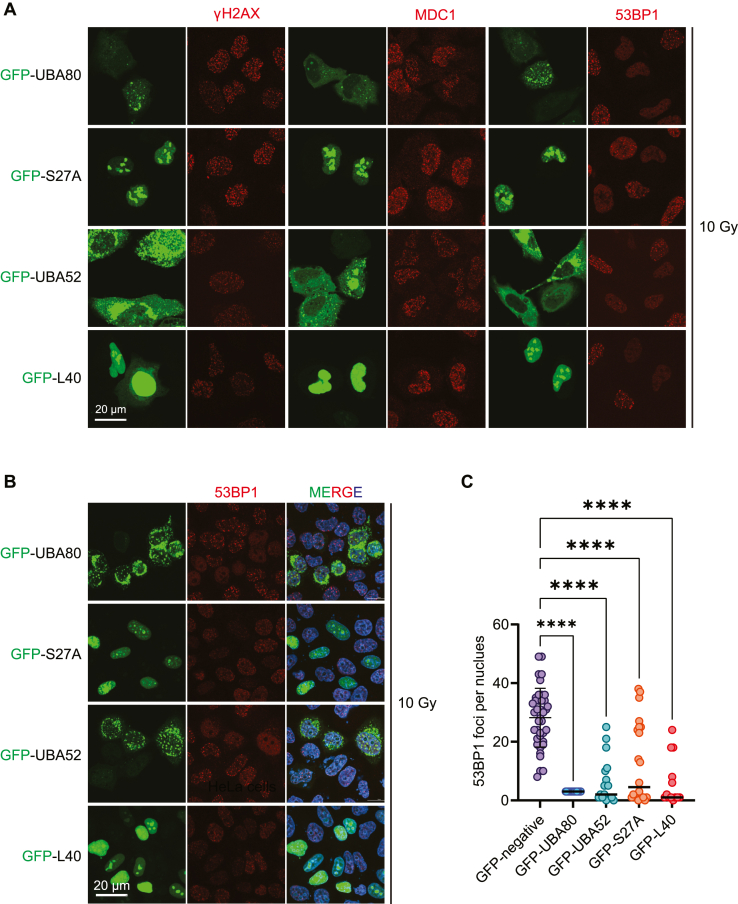
**UBA80 and UBA52 regulate 53BP1 accrual upon DNA damage.***A* and *B,* ectopic expression of UBA80, UBA52, S27A, and L40 suppress 53BP1 ionizing radiation–induced foci. U2OS cells (*A*) and HeLa cells (*B*) were transfected with GFP-expression plasmids as indicated. Twenty four hours after transfection, cells were treated with 10 Gy and recovered for 1 h, followed by immunofluorescence analysis using indicated antibodies. *C,* quantification of 53BP1 ionizing radiation–induced foci in HeLa cells as in B. Data presented as mean±SD. One-way ANOVA was used for statistical analysis ∗∗∗∗*p < 0.0001*.

To investigate mechanistically how UBA80 and UBA52 regulate DNA repair, we ectopically overexpressed GFP-UBA80, GFP-S27A, GFP-UBA52, and GFP-L40 in U2OS cells. We found that 53BP1 IRIF is drastically impaired, without discernible difference in γH2AX and MDC1 IRIF (Fig. 4*A*). These data suggest that UBA80 and UBA52 may negatively regulate 53BP1 recruitment to DNA damage sites downstream of γH2AX and MDC1. (Fig. 4*A*). We also observed a consistent UBA80, S27A, UBA52, and L40 overexpression–mediated 53BP1 IRIF impairment in HeLa cells, confirming this defect is a genetic attribution by UBA80 and UBA52 (Fig. 4, *B* and *C*).

### UBA80 and UBA52 inhibit RNF168-mediated ubiquitination of H2A/H2AX on K13/15

**Figure 5:**
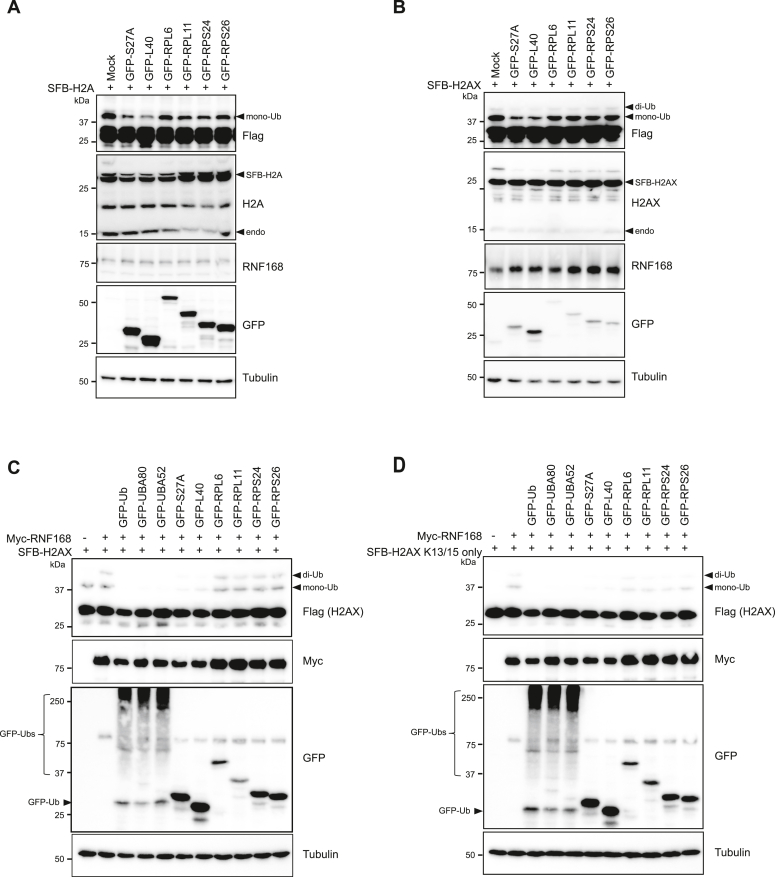
**UBA80 and UBA52 regulate RNF168-mediated H2A(X) ubiquitination.***A* and *B,* S27A and L40 inhibit H2A(X) ubiquitination. HEK293T cells were cotransfected with SFB-H2A (*A*) or SFB-H2AX (*B*) and indicated GFP-tagged ribosomal proteins. After 24 h, cells were harvested with 1× Laemmli sample buffer, followed by Western blot analysis with indicated antibodies. Similar results over three independent experiments. *C* and *D,* S27A and L40 suppress RNF168-mediated H2A(X) ubiquitination at K13/15. HEK293T cells were cotransfected with SFB-H2AX (*C*) or SFB-H2AX K13/15 only (*D*) with Myc-RNF168 and GFP-tagged ribosomal proteins as indicated. Cells were harvested with a 1× Laemmli sample buffer 24 h later, followed by Western blot analysis. SFB, S-protein-FLAG–Streptavidin-binding peptide.

Given that UBA80 and UBA52 bind to both RNF168 and H2A/H2AX and suppress 53BP1 accrual at DNA lesion, we hypothesized that UBA80 and UBA52 are involved in regulating RNF168-mediated H2A/H2AX ubiquitination, which acts upstream of 53BP1 recruitment to damaged chromatin. To test this, we cotransfected SFB-H2A or SFB-H2AX with GFP-S27A, GFP-L40, or other ribosomal proteins, including RPL6, RPL11, RPS24, and RPS26, as controls. Intriguingly, GFP-S27A and GFP-L40, but not other ribosomal proteins, reduced H2A and H2AX ubiquitination without altering the endogenous RNF168 protein level (Fig. 5, *A* and *B*).

To further validate the specificity of UBA80 and UBA52 in the regulation of RNF168-mediated ubiquitination, we co-expressed Myc-RNF168 and SFB-H2AX or SFB-H2AX-K13/15 only in the presence of different ribosomal proteins. We found that UBA80, UBA52, S27A, and L40, but not RPL6, RPL11, RPS24, and RPS26, were able to inhibit the RNF168-specific H2AX ubiquitination at K13 and K15 residues (Fig. 5, *C* and *D*) and internal ubiquitination of UBA80 was dispensable for RNF168 inhibition (Fig. S4*A*). Consistently, the depletion of UBA80 and UBA52 showed an increase in RNF168-mediated H2AX ubiquitination (Fig. S4*B*). Together, our data suggest that UBA80 and UBA52 are intrinsic suppressors of the RNF168 function.

### S27A and L40 bind to H2A(X) and RNF168 to fine-tune DNA repair

**Figure 6:**
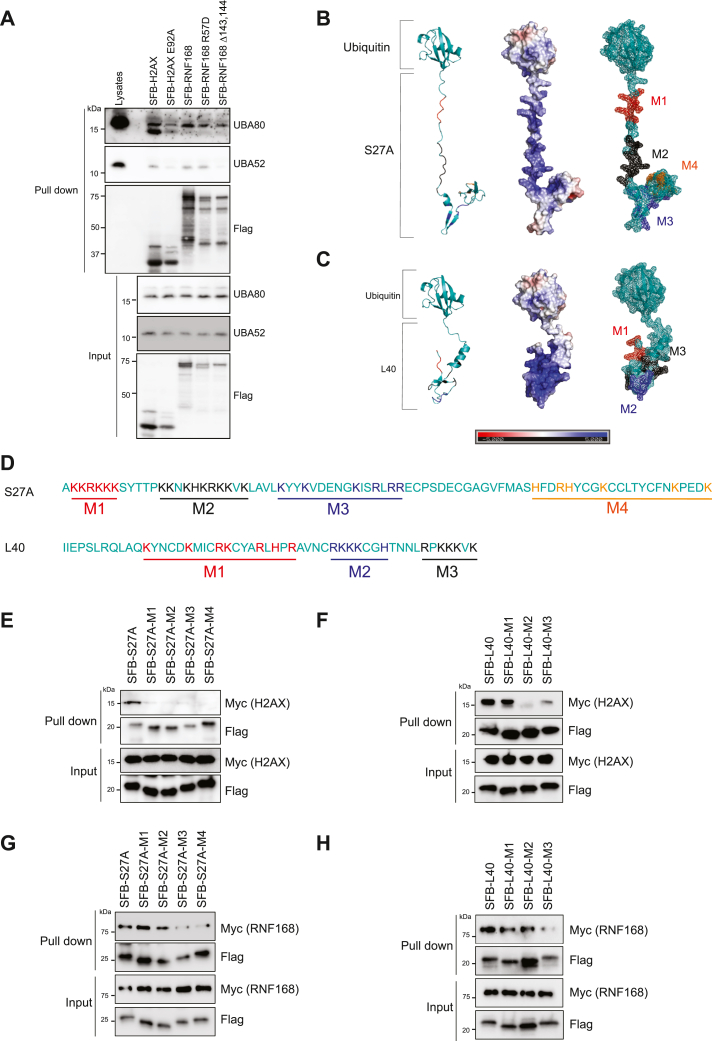
**UBA80 and UBA52 interact with H2A(X) and RNF168.***A,* UBA80 and UBA52 interact with H2A(X) and RNF168 through the nucleosome acidic residues. HEK293T cells were transfected with indicated SFB-expressing vectors. Streptavidin pull-down of SFB-tagged proteins followed by Western blotting analysis. *B* and *C,* AlphaFold predicted structure of UBA80 and UBA52 are presented as cartoon (*left*), space-filling electrostatic potential distribution (*middle*), and mesh with color-labeled positively charged clusters (*right*). *D,* S27A and L40 clustered mutations used in pull-down assay for protein–protein interaction. Color annotated residues were mutated to alanine (*E*–*H*). Mapping S27A and L40 interacting interface with H2A(X) and RNF168. *E* and *F,* Myc-H2AX was cotransfected with SFB-S27A or SFBL40 mutants in HEK293T cells. *G* and *H,* Myc-RNF168 was cotransfected with SFB-S27A or SFBL40 mutants in HEK293T cells, followed by Streptavidin pull-down and Western blotting analysis using indicated antibodies.

Previous studies identified the nucleosome acidic patch, RNF168 arginine anchor, and E143/E144 acidic region within the UMI domain as the key molecular entities directing the RNF168-mediated H2A/H2AX site–specific ubiquitination (13). As UBA80 interacts with the central acidic domain of MDM2 (34), we hypothesized that UBA80 and UBA52 interact with H2AX/H2AX and RNF168 through their acidic residues. By pull-down assay, H2AX E92A acidic patch and RNF168 E143/E144 mutants showed a reduction in both UBA80 and UBA52 binding (Fig. 6*A*). To further pinpoint how S27A and L40 interact with the acidic residues of RNF168 and the nucleosome, we analyzed their electrostatic potential. We found that both S27A and L40 are highly positively charged (Fig. 6, *B* and *C*). To systematically map the binding region for RNF168 and the nucleosome, we generated alanine mutations based on their structural clustering (Fig. 6, *B*–*D*). Surprisingly, pull-down experiments showed that every S27A cluster mutation abolished its interaction with H2AX, while M3 and M4 mutants showed reduced binding affinity to RNF168 (Fig. 6, *E* and *F*). For L40, M2 mutant largely abolished its interaction with H2AX while the M3 mutant showed significantly reduced interaction with RNF168 with a modest reduction in H2AX interaction (Fig. 6, *G* and *H*). These data suggest that both S27A and L40 have distinctive molecular modes of action in masking RNF168 and the nucleosome acidic patch.

**Figure 7:**
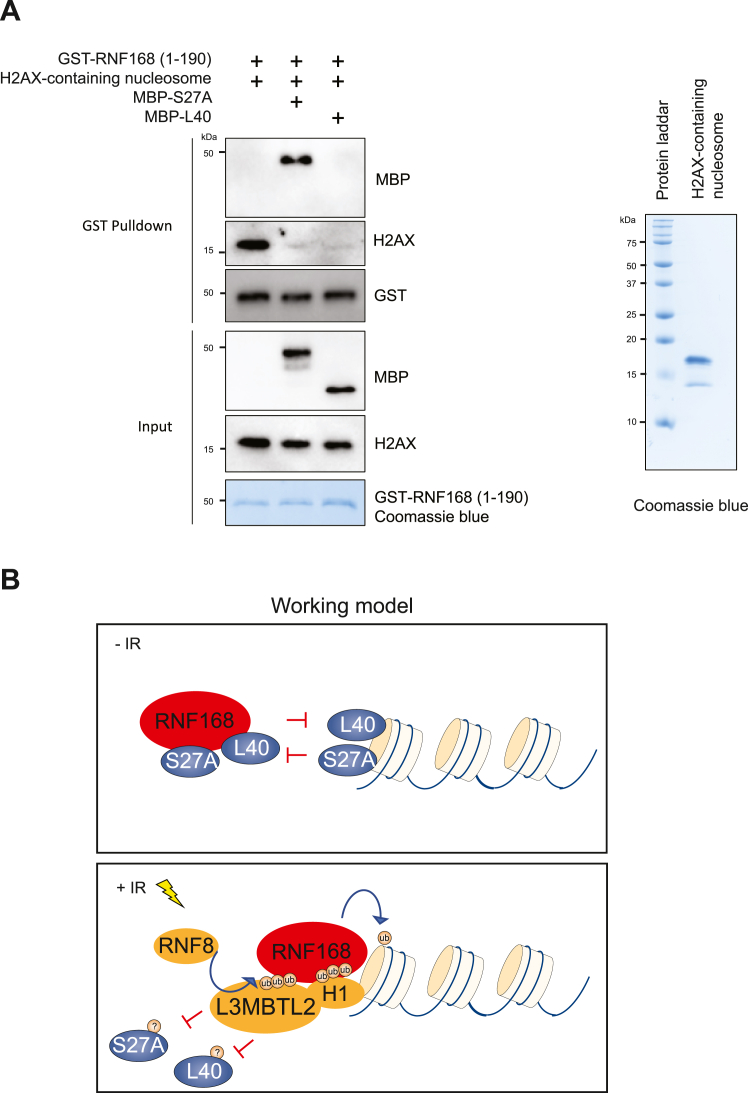
**S27A and L40 suppress RNF168-nucleosome engagement.***A,* RNF168-nucleosome binding is inhibited by S27A and L40 *in vitro*. GST pull-down assay using *in vitro* purified proteins and recombinant H2AX-containing nucleosome (*left*), and recombinant H2AX-containing nucleosome was stained using Coomassie blue. *B,* schematic diagram of the proposed model for the action of S27A and L40 in RNF168-mediated H2A(X) ubiquitination upon DNA damage. GST, glutathione *S*-transferase.

To provide direct evidence that S27A and L40 negatively regulate the RNF168-nucleosome engagement, we performed a competitive *in vitro* pull-down assay using purified H2AX-containing nucleosome and glutathione *S*-transferase (GST)-RNF168 (a.a.1-190), which is sufficient for its docking onto the nucleosome. Strikingly, the GST-pull down for H2AX is drastically reduced in the presence of S27A or L40 (Fig. 7*A*), suggesting that S27A and L40 are competing with the interaction between RNF168 and the nucleosome.

Furthermore, ectopic expression of GFP-S27A and GFP-L40 in U2OS cells showed slower γH2AX foci resolving rate after a low dose of irradiation (Fig. S5, *A*–*C*), suggesting that DNA breaks persist in the S27A- and L40-overexpressing cells, potentially due to impaired 53BP1 IRIF. Collectively, our data provide evidence that UBA80 and UBA52 interact and mask the RNF168–nucleosome interaction and fine-tune the chromatin-mediated DNA repair *via* sequestering the ubiquitination signaling cascade (Fig. 7*B*).

## Discussion

The RNF168-mediated histone H2A ubiquitination is crucial in recruiting DNA repair proteins to damaged chromatin. The upstream genetic factors that promote RNF168 and the functional domains have been characterized extensively (11, 22). The RNF168-damaged chromatin recruitment is primarily mediated by the two motif-interacting-with ubiquitin (15, 18, 21). Recent studies have highlighted how RNF168 is molecularly regulated to achieve target specificity through its arginine anchor–nucleosome acidic patch and the UMI acidic residues E143/E144-H2A alpha1-extension helix (13, 23, 24, 25, 35). It is clear that there are multiple layers of regulation for RNF168-mediated ubiquitination to ensure the proper recruitment and removal of repair proteins at damaged chromatin.

Upstream regulation of RNF168 includes maintaining the RNF168 protein level by TRIP12 and UBR5, thereby controlling the ubiquitin signaling spreading (36). Additionally, RNF168-mediated ubiquitination can be counteracted by the removal of H2A ubiquitination marks by deubiquitinases, including USP3, USP16, USP44, and USP51 (37, 38, 39, 40, 41, 42, 43), and competitive binding by RNF169 to the H2A ubiquitination that is mediated by RNF168 (18, 44, 45, 46). Here, we identify UBA80 and UBA52 as regulatory proteins involved in fine-tuning the ubiquitin DDR signaling. Mechanistically, UBA80 and UBA52 bind to the regulatory acidic E143/E144 residues of RNF168 to suppress its substrate targeting. In parallel, they also interact with the H2A acidic patch. Their interactions, at least in part, are negatively regulated by DNA damage. It is unclear how IR disrupts their interaction. We speculate that it could be regulated by the posttranslational modifications of the ribosomal proteins. It is also possible that the RNF168 binding is outcompeted by the ubiquitinated RNF8 substrates upon damage (31, 47, 48, 49). Their dissociation may promote the spatial engagement and proper orientation between RNF168 and nucleosome to the target residues. We demonstrate that UBA80 and UBA52 function as molecular harnesses for RNF168 substrate targeting *via* specific interaction under physiological conditions.

In mammalian cells, ubiquitin is synthesized from three types of precursor proteins, UBA, UBB, and UBC are polymers of ubiquitin, which contribute to the intracellular content of ubiquitin at the basal level (50). UBA is encoded by two genes, UBA80, and UBA52, which is a single ubiquitin carboxyl terminally fused to a ribosomal protein. The UBA is cotranslationally and posttranslationally cleaved into individual ubiquitin moieties and ribosomal proteins S27A and L40 by UCHL1, UCHL3, USP7, and USP9X (29, 51). The UBA genes are constitutively expressed and contribute to the basal cellular ubiquitin pool along with the UBB and UBC genes, which are transcriptionally upregulated in response to stress response including DNA damage, oxidative stress, and proteasome inhibition (50, 52, 53, 54). While it is unclear how long endogenous UBA80 and UBA52 remain in precursor form after biogenesis, the cleavage of the UBA80 and UBA52 is essential for their ribosomal subunit maturation and protein synthesis (30, 31). A study showed that UBA80 and UBA52 transfection in cells displayed cleavage predominantly (28). We believe that it largely reflects the endogenous proteins, which will make the S27A and L40 ribosomal subunit readily available for the ribosomal machinery assembly. Collectively, emerging evidence showed that UBA80 and UBA52 are involved in cellular stress. However, the cotranslational processing and regulation of UBA proteins and the functions of their C-terminal ribosomal proteins in DNA damage are largely unclear.

Apoptotic stress induces the expression of UBA80 and UBA52, which correlates to histone H2A ubiquitination reduction during apoptosis (28). Consistently, our data demonstrate a potential mechanism by which S27A and L40 suppress H2A(X) ubiquitination through competitive interactions with the nucleosome acidic patch and the RNF168 acidic region and inhibit RNF168-targeting efficiency. Interestingly, UBA80 and UBA52 transcription is increased in a p53-dependent manner within 24 h after treatment of DNA-damaging agents, such as etoposide, methyl methanesulfonate, and UV (50, 55). Contrarily, a recent study showed that cas9-induced DNA double-strand breaks lead to UBA80 protein level reduction in later time points *via* proteasomal degradation independent of p53 signaling (56), highlighting the complexity of regulation of UBA80 in response to DNA damage. A detailed systematic investigation is needed to further elucidate the regulation and functions of these ub-ribosomal protein precursors in response to different types of genotoxic insults.

Notably, both ribosomal proteins, S27A and L40, have a high proportion of basic residues (Fig. 6, *B* and *C*) (57), which potentially attributes to the binding to the H2A acidic patch and RNF168 acidic residues E143/E144. Our data showed that they seem to utilize a different binding interface to interact with the nucleosome and RNF168. Unexpectedly, for S27A, all cluster mutants showed a drastic reduction in binding affinity to H2AX, while mutants M3 and M4 showed reduced binding affinity to RNF168. As M3 and M4 reside on the highly structured region of the S27A, we speculate that mutations of these two positively charged clusters may alter the protein structure conformation that is critical for both H2A/X and RNF168 binding. For H2A/X, additional residues may be required within the M1 and M2 clusters for interaction or orientation. For L40, M2 and M3 showed reduced binding affinity to H2A/X, while M3 showed significantly weaker binding to RNF168. Similar to S27A, we believe that L40 may have more than one binding residue with the nucleosome or the mutant could potentially disrupt the structural interface that is required for the interaction. It is also possible that highly basic proteins, like S27A and L40, have a tendency to interact with nucleosomal DNA, which may also affect the interaction with the nucleosome (28). Although we have narrowed down some of the potential regions for RNF168 interactions, due to the complexity of their interaction, systematic mutagenesis may not be the best approach to definitively map the molecular interaction between these proteins. Follow-up structural study would be more appropriate to definitively map their binding interface and visualize the dynamics of their molecular interactions.

*In vitro*, competitive GST pull-down assay demonstrated that, biochemically, S27A can displace the nucleosome for RNF168 interaction. Interestingly, although there was a drastic reduction in the RNF168–nucleosome interaction in the presence of L40, no discernable L40 protein was detected in the GST pull-down sample. We suspect that L40 may have a preferential affinity to the nucleosome over RNF168. Mechanistically, these data provide strong evidence that both S27A and L40 are involved in regulating the RNF168-nucleosome engagement. In line with the interaction experiments, ectopic expression of UBA80, UBA52, S27A, or L40 hinders RNF168-dependent ubiquitination and 53BP1 foci formation (Figs. 4 and 5). These ribosomal proteins of two ubiquitin precursors serve as intrinsic inhibitors for RNF168-mediated H2A(X) ubiquitination by harnessing their engagement to fine-tune the signaling pathway. The interactions between the UBA proteins and RNF168 or H2A(X) are dissociated upon DNA damage, which allows RNF168-nucleosome binding and H2A(X) ubiquitination catalysis.

In the current study, we were unable to generate KO cells for UBA80 and UBA52. The siRNA-mediated UBA80 and UBA52 knockdown showed a drastic reduction in cell proliferation (Fig. S3*F*). Consistent with a previous report (30, 32, 33), cells with UBA80 or UBA52 depletion exhibit abnormal cell cycle profiles (Fig. S3*E*). Since *de novo* synthesis of ubiquitin is largely contributed by UBB and UBC and free ubiquitin levels in cells are maintained by recycling from the target substrates (50), the cell viability is likely due to the perturbation of ribosomal machinery in UBA80- and UBA52-depleted cells. The essential ribosomal functions for UBA80 and UBA52 also limit our study in interpreting the observations in DNA repair defects, which can be indirectly affected by perturbed cell proliferation and dysregulated cell cycle.

We found that cells with UBA80 and UBA52 depletion exhibit more γH2AX, MDC1, and 53BP1, which is due to delayed or unrepaired DNA breaks. However, the increased 53BP1 foci can also be affected by the enhanced RNF168-mediated H2A(X) ubiquitination in the absence of UBA80 and UBA52 which is in line with the reduced H2A(X) ubiquitination and 53BP1 foci in UBA80, UBA52, S27A and L40 overexpressed cells. Interestingly, S27A and L40 are recruited to laser-induced microirradiation and the recruitment is RNF168-dependent. We speculate that their damaged chromatin recruitment is not RNF168 catalytic activity–dependent due to their inhibitory nature to RNF168-mediated ubiquitination. Moreover, their recruitment is relatively weaker than RNF168 and 53BP1, suggesting that they may localize to damaged sites with the initial recruitment of RNF168 *via* basal physical interaction.

Clinically, it has been reported that UBA80 is overexpressed in solid tumors including kidney, breast, cervical, and colon cancers and chronic myeloid leukemia. It is pathologically associated with increased proliferation, regulating cell cycle progression, and inhibiting apoptosis (33, 58, 59). In addition, it is recently reported that the expression of UBA80 was upregulated in lung adenocarcinoma cells and correlated with lung adenocarcinoma progression and poor prognosis (32).

Many ribosomal proteins have extraribosomal functions but only a few ribosomal proteins have been demonstrated to directly participate in regulating the DDR pathway (60). Similar to UBA80 and UBA52, RPL6 is also recruited to DNA damage sites *via* H2A interaction in a damage-dependent manner. It acts upstream of MDC1 and is required for G2-M checkpoint and cell survival in response to DNA damage (61). Another ribosomal protein, RPS27L, plays a multifaceted role in maintaining genome stability *via* competitive binding with the p53–MDM2 complex, modilating the MRE11A/RAD50/NBS1–ATM signal, and interacting with the FANCD2–FANCI complex (62, 63, 64, 65). Other ribosomal proteins including RPS9, RPS3, and RPP0 were also found to be involved in DNA repair in other organisms (60), highlighting potential unexplored ribosomal proteins with extraribosomal functions.

Overall, our study provides additional evidence on extraribosomal functions of ribosomal proteins and expands our knowledge in understanding the refined regulation of the chromatin-based DDR pathway. We present a novel mechanism that UBA80 and UBA52 fine-tune the RNF168 signaling by masking the substrate targeting the regulatory acidic region. Our finding might also help identify molecular targets to kill cancer cells by exploiting the DDR pathway.

## Experimental procedures

### Cell culture

HEK293T, U2OS, and HeLa cells were purchased from American Type Culture Collection and cultured in Dulbecco’s modified Eagle medium with 10% fetal bovine serum supplemented with 100 U/ml penicillin and 100 μg/ml streptomycin at 37 °C and 5% CO_2_. Transfections were carried out using PEI (Polysciences) according to the manufacturer’s instructions.

### Plasmids and siRNAs

Human UBA80 and UBA52 plasmids were purchased from Addgene (pET23a-HsRPS27a, pH0103_UBA52_Ubiquitin) and were subcloned into Gateway-compatible destination vectors using Gateway cloning technology (Invitrogen). RNF168 (a.a.1-190) was cloned into GST-expression vector using gateway system. Mutations were created by the Q5 Site-Directed Mutagenesis (New England Biolabs) according to the manufacturer's instructions. Mutagenesis primers were obtained through Integrated DNA Technologies. S27A and L40 mutant gene fragments were synthesized by Integrated DNA Technologies and subcloned into Gateway cloning system. The open reading frame and mutagenesis were verified by Sanger sequencing. siRNA SMARTpools for UBA80 and UBA52 and ON-TARGETplus Nontargeting siRNA were purchased from Dharmacon.

### Antibodies

Primary antibodies used in this study were UBA80 (Raybiotech, 144-02027-50), UBA52 (Bio-Rad, VPA00424), Flag M2 (Sigma, F1804), Myc (Santa Cruz, sc-40), GFP (Invitrogen, A11122), 53BP1 (Novus Biologicals, NB100-304), BRCA1 (Santa Cruz Biotechnology, SC-6954), γH2AX (Millipore, 05-636), H2AX (Cell Signaling, 2595S), H2A (Cell Signaling, 2578), tubulin (Abcam, ab6046), MDC1 (Abcam, ab11169), RNF168 (Sigma-Aldrich, ABE367), GST (Millipore, 71007-3), and MBP (Abcam, ab119994). For Western blotting, secondary antibodies—horseradish peroxidase-linked anti-rabbit immunoglobulin G and horseradish peroxidase-linked anti-mouse immunoglobulin G—were purchased from Cell Signaling (0704 and 0706). For immunofluorescence, Alexa Fluor 488 goat anti-rabbit and Alexa Fluor 594 goat anti-mouse antibodies were used (Invitrogen).

### Tandem affinity purification

Tandem affinity purification on chromatin was performed as previously described. RNF168 was subcloned into pMH-SFB (Addgene ID: 99391) to drive mammalian expression of SFB-tagged RNF168 proteins. Briefly, SFB-RNF168–transfected HEK293T cells were harvested with NETN buffer (150 mM NaCl, 0.5 mM EDTA, 20 mM Tris–HCl at pH 8.0, 0.5% NP-40) with protease inhibitors for 10 min at 4 °C. The supernatant was discarded, and the pellet was washed with NETN buffer and digested again with NETN buffer with Turbonuclease (Sigma-Aldrich) to obtain the chromatin-bound fraction for 1 h at 4 °C. After centrifugation, the chromatin cell lysate was incubated with Streptavidin Sepharose (GE Healthcare) overnight, followed by washing with NETN buffer three times and eluted with 2 mM biotin at 4 °C. The eluent was then incubated with S-protein beads (EMD Millipore) overnight, washed with NETN buffer three times, and eluted with 1 × Laemmli buffer. The immuno-complex was subjected to SDS-PAGE and excised for mass spectrometry analysis.

### Streptavidin pull-down assay and Western blotting

Cells were transfected with SFB-fused proteins as indicated and harvested with a NETN buffer with Turbonuclease at 4 °C for 1 h. The lysates were incubated with Streptavidin beads for 1 h at 4 °C, followed by washing with NETN buffer four times. The immunoprecipitated complexes were eluted with 1 × Laemmli buffer and were resolved by SDS-PAGE, transferred to polyvinylidene difluoride membranes, immunoblotted with antibodies as indicated, and imaged using Bio-Rad ChemiDoc MP.

### Immunofluorescence and confocal microscopy

Cells were seeded on poly-L-lysine–coated coverslips (BD biosciences) 24 hours before the transfection or the experiment. Coverslips were washed in PBS and fixed in 3% paraformaldehyde for 10 min at room temperature, followed by permeabilization with 0.5% Triton X-100 solution for 5 min. Samples were incubated with primary antibodies in 3% bovine serum albumin for 1 h, washed, and incubated with secondary antibodies for 1 h without exposure to light, followed by incubation with 4′,6-diamidino-2-phenylindole (200 μg/ml) for 10 min at room temperature. Samples were then mounted onto glass slides with an antifade solution (0.02% p-phenylenediamine [Sigma, P6001] in 90% glycerol in PBS). Samples were visualized and captured using a Ti-2 inverted C2 + confocal microscope.

### Laser-induced micro-irradiation

U2OS cells were transfected with GFP-expression vectors as indicated. Twenty four hours prior to the experiment, cells were seeded on 35 mm glass-bottom dishes. Laser-induced micro-irradiation was performed using a Nikon Ti-2 inverted fluorescent microscope and C2 + confocal system. Cells were damaged with a fixed-wavelength (405 nm) laser at 60% power. Live-cell images were recorded in 1-min intervals after damage.

### Cell cycle analysis

For cell cycle analysis, cells were irradiated with 3 Gy and incubated for the indicated time. Cells were trypsinized and fixed in 80% ethanol for 15 min at 4 °C. Cells were incubated with propidium iodide (40 μg/ml)/RNase A (4 μg/ml) in Tris-EDTA buffer at 37 °C for 30 min. Samples were analyzed by BD Accuri C6 Plus flow cytometry.

### Colony formation survival assays

Cells were seeded at a density of 1000 cells per well in a 6-well plate in triplicate. At 14 days, cells were fixed and stained with Coomassie blue staining solution, washed, dried, and followed by manual counting of visible colonies.

### *In vitro* GST pull-down assay

GST-RNF168 (a.a.1-190), MBP-S27A, and MBP-L40 were expressed in BL21 and purified using glutathione sepharose (GE Health) and amylose resin (NEB). MBP-S27A and MBP-L40 were eluted using 10 mM maltose in 1XPBS. Recombinant H2AX–containing nucleosome was purchased from EpiCypher. *In vitro* pull-down assay was performed as previously described (17). Briefly, GST-RNF168 (a.a.1-190) was immobilized on GST agarose with binding buffer (50 mM Tris–HCl pH8.0, 150 mM NaCl, 0.95 NP-40, 0.1% bovine serum albumin), 3 μg of nucleosome was added with or without MBP-S27A and MBP-L40 and incubated for 2 h at 4 °C. The pull-down reactions were then washed with the binding buffer, followed by eluting in Laemmli SDS-PAGE sample buffer for Western blotting analysis.

### DNA repair kinetics analysis

U2OS-expressing GFP-S27A and GFP-L40 were treated with a low dose of X-ray (2 Gy for immunofluorescence and 4 Gy for Western blot analysis) Cells were then harvested at the indicated time points, followed by immunofluorescence and Western blotting analyses. γH2AX was used as a DNA damage marker for repair kinetics quantification.

### Molecular graphics

Molecular graphics were generated using PyMOL (https://pymol.org/2/). UBA80 and UBA52 protein structures were obtained from the AlphaFold protein structure database. Electrostatic potential was calculated by Adaptive Poisson Boltzmann Solver.

## Data availability

The experimental data sets and materials generated and analyzed during the current study are available from the corresponding author upon request. Proteomic data have been deposited to Proteome Xchange (accession: PXD037840).

## Supporting information

This article contains supporting information.

## Conflict of interest

The authors declare that they have no conflicts of interest with the content of this article.
