# Supplementary material for: UBA80 and UBA52 fine-tune RNF168-dependent histone ubiquitination and DNA repair
Source: J Biol Chem. 2023 Jul 13;299(8):105043. doi: 10.1016/j.jbc.2023.105043 (PMC10413357; doi:10.1016/j.jbc.2023.105043)
Supplement: Supporting information [file mmc1.pdf]

UBA80 and UBA52 fine-tune RNF168-dependent histone ubiquitination and DNA repair

Seong-Ok Lee[1,2], Jessica L Kelliher[2,3], Wan Song[4], Kyle Tengler[4], Aradhan Sarkar[4,] Eloise Dray[5], Justin W C Leung* [2,4]

[1]Department of Pharmacology and Toxicology, College of Medicine, University of Arkansas for Medical Sciences, Little Rock, AR, USA.

[2]Department of Radiation Oncology, College of Medicine, University of Arkansas for Medical Sciences, Little Rock, AR, USA.

[3]Department of Biochemistry and Molecular Biology, College of Medicine, University of Arkansas for Medical Sciences, Little Rock, AR, USA.

[4]Department of Radiation Oncology, University of Texas Health Science Center at San Antonio, San Antonio, TX, USA.

[5]Department of Biochemistry and Structural Biology, University of Texas Health Science Center at San Antonio, San Antonio, TX, USA.

- Correspondence to Justin W. Leung

Email: Leungj@uthscsa.edu

Supporting information

Figure S1. Ubiquitin-conjugated ribosomal proteins are chromatin-interacting proteins. (A) Schematic diagram of tandem affinity purification and fractionation. Chromatin fraction of RNF168 and interacting partners were obtained using turbonuclease and chromatin extraction buffer after pre-extracted with NETN buffer. (B) Schematic diagram of four human ubiquitin precursor genes.

Figure S2. UBA80 and UBA52 are co-translationally and post-translationally processed. (A) Schematic illustration of GFP-expression vectors for UBA80, UBA52used in the experiments. (B and C) UBA80 and UBA52 are precursor proteins for ubiquitin and ribosomal protein. HEK293T cells were transfected with indicated GFP-tagged constructs. After 24 h, cells were harvested in a 1x Laemmli sample buffer, followed by immunoblotting analysis using the indicated antibodies. (D) UBA80 and UBA52 precursor proteins are not recruited to DNA damage sites. U2OS cells with GFP-UBA80 and UBA52 CR mutants were treated with laser-induced micro-irradiation and analyzed using confocal microscope. Red arrows indicate the laser path. (E) UBA80 and UBA52 are not RNF168 substrates. HEK293T cells were co-transfected with SFB-H2AX, GFP-S27A, or GFP-L40 with Myc-RNF168. After 24 h, cells were harvested and immunoblotted using indicated antibodies. (F) DNA damage does not induce UBA80 or UBA52 ubiquitination. HEK293T cells were irradiated with 5 Gy or 10 Gy, incubated as indicated, and then harvested in 1x Laemmli sample buffer, followed by western blot analysis.

Figure S3. Depletion of UBA80 and UBA52 regulates the cell cycle and cell proliferation. (A) UBA80 and UBA52 depletion do not alter ubiquitin levels in cells. U2OS cells were harvested 72 h after siRNA transfection, followed by western blot analysis with indicated antibodies. (B) UBA80 and UBA52 depletion impair DNA repair protein accumulation at DNA breaks. U2OS cells were transfected with indicated siRNAs. After 72 h, cells were irradiated with 3 Gy and fixed as indicated timepoints, then followed by immunofluorescence analysis using MDC1 and BRCA1

antibodies (C and D). Quantification of nuclear MDC1 (C) and BRCA1 (D) foci as in B. Each dot represents a single cell. For each condition, images containing at least 100 cells were acquired. Data presented as mean±SD. Two-way ANOVA was used for statistical analysis. (E) UBA80 and UBA52 depletion lead to cell cycle defects. DNA content of cells was measured by PI staining. (F) UBA80 and UBA52 depletion impair cell proliferation. Representative pictures of colony formation assay and quantification of colony numbers.

Figure S4. UBA80 and UBA52 suppress RNF168-mediated H2AX ubiquitination. (A) Internal ubiquitination of UBA80 is dispensable for RNF168-mediated H2AX ubiquitination inhibition. HEK293T cells were co-transfected with SFB-H2AX and Myc-RNF168 with GFP-UBA80 or GFP-UBA80 K113R. After 24 h, cells were harvested with 1x Laemmli sample buffer, followed by western blot analysis with indicated antibodies. (B) HEK293T cells were transfected with indicated siRNAs. After 72 h, cells were cotransfected as indicated.

Figure S5. Ectopic expression of S27A and L40 impairs DNA repair kinetics. (A) U2OS cells with GFP-S27A or GFP-L40 overexpression were treated with 2 Gy. Cells were fixed at indicated time points and stained with γH2AX antibody. (B) quantification of γH2AX foci number as in A. Data presented as mean±SD. Two-way ANOVA was used for statistical analysis. *<0.05; ****$P<0.0001$ (C) U2OS cells were transfected with SFB-S27A and SFB-L40 were treated with 4 Gy and harvested at indicated timepoint followed by western blot analysis using γH2AX antibody.

Figure S1

A

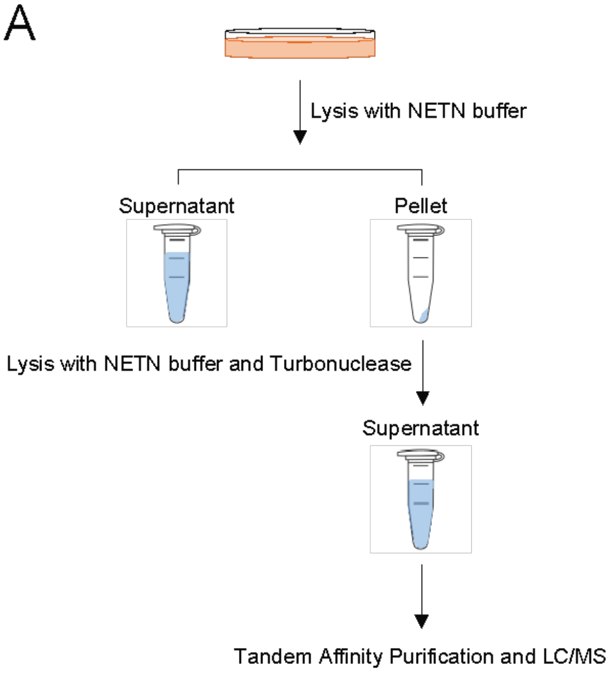

Lysis with NETN buffer
Supernatant
Pellet
Lysis with NETN buffer and Turbonuclease
Supernatant
Tandem Affinity Purification and LC/MS

B

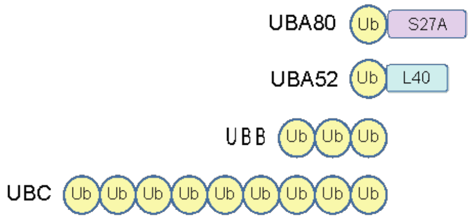

UBA80
Ub
S27A
UBA52
Ub
L40
UBB
Ub Ub Ub
UBC
Ub Ub Ub Ub Ub Ub Ub Ub Ub

Figure S2

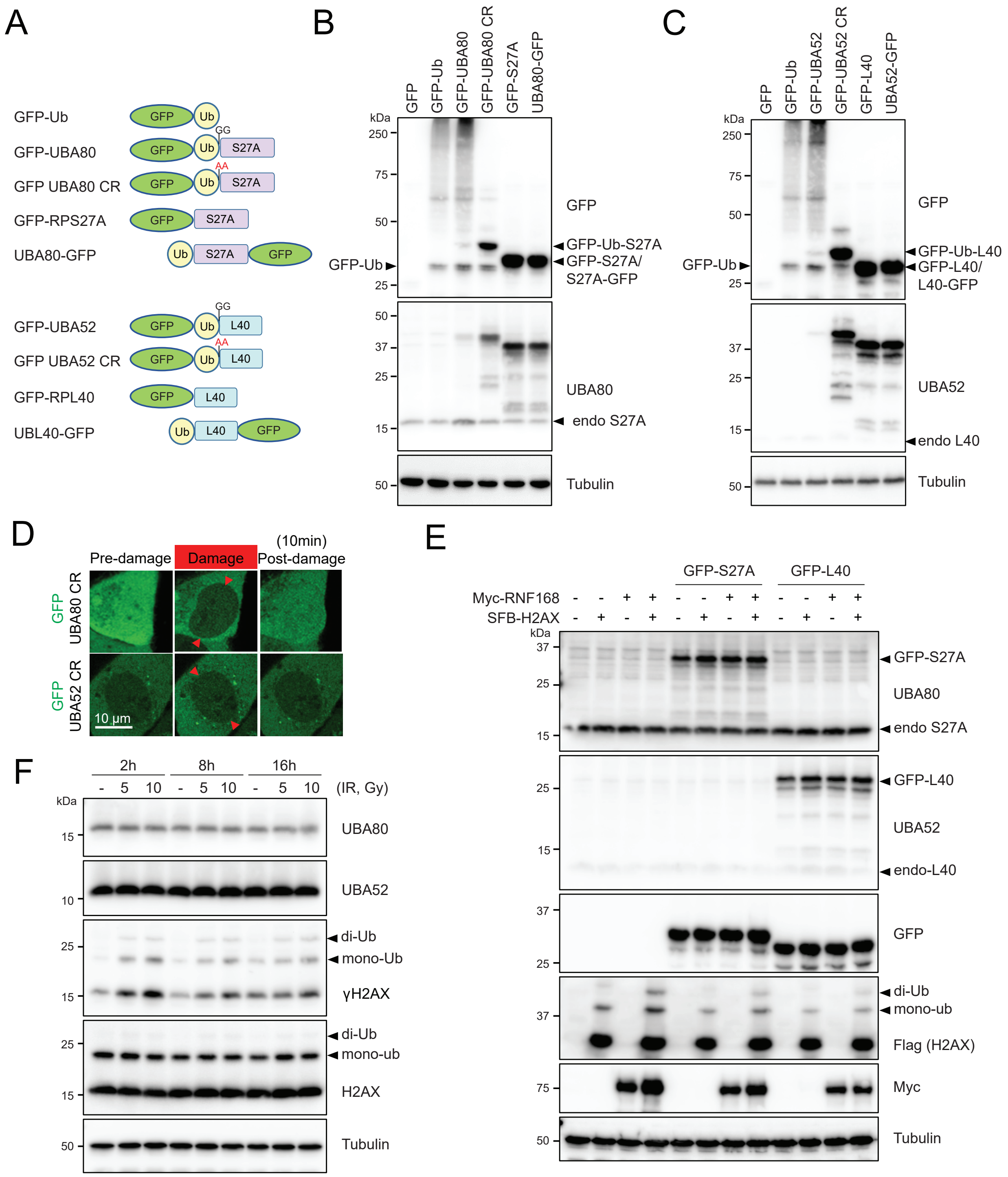

A
GFP-Ub
GFP-UBA80
GFP UBA80 CR
GFP-RPS27A
UBA80-GFP
GFP-UBA52
GFP UBA52 CR
GFP-RPL40
UBL40-GFP
GFP
Ub
S27A
L40
GG
AA
B
GFP
GFP-Ub
GFP-UBA80
GFP-UBA80 CR
GFP-S27A
UBA80-GFP
kDa
GFP-Ub-S27A
GFP-S27A/
S27A-GFP
UBA80
endo S27A
Tubulin
C
GFP-UBA52
GFP-UBA52 CR
GFP-L40
UBA52-GFP
GFP-Ub-L40
GFP-L40/
L40-GFP
UBA52
endo L40
D
Pre-damage
Damage
(10min)
Post-damage
GFP UBA80 CR
GFP UBA52 CR
10 μm
E
GFP-S27A
GFP-L40
Myc-RNF168
SFB-H2AX
endo-L40
di-Ub
mono-ub
Flag (H2AX)
Myc
F
2h
8h
16h
(IR, Gy)
mono-Ub
γH2AX
H2AX

Figure S3

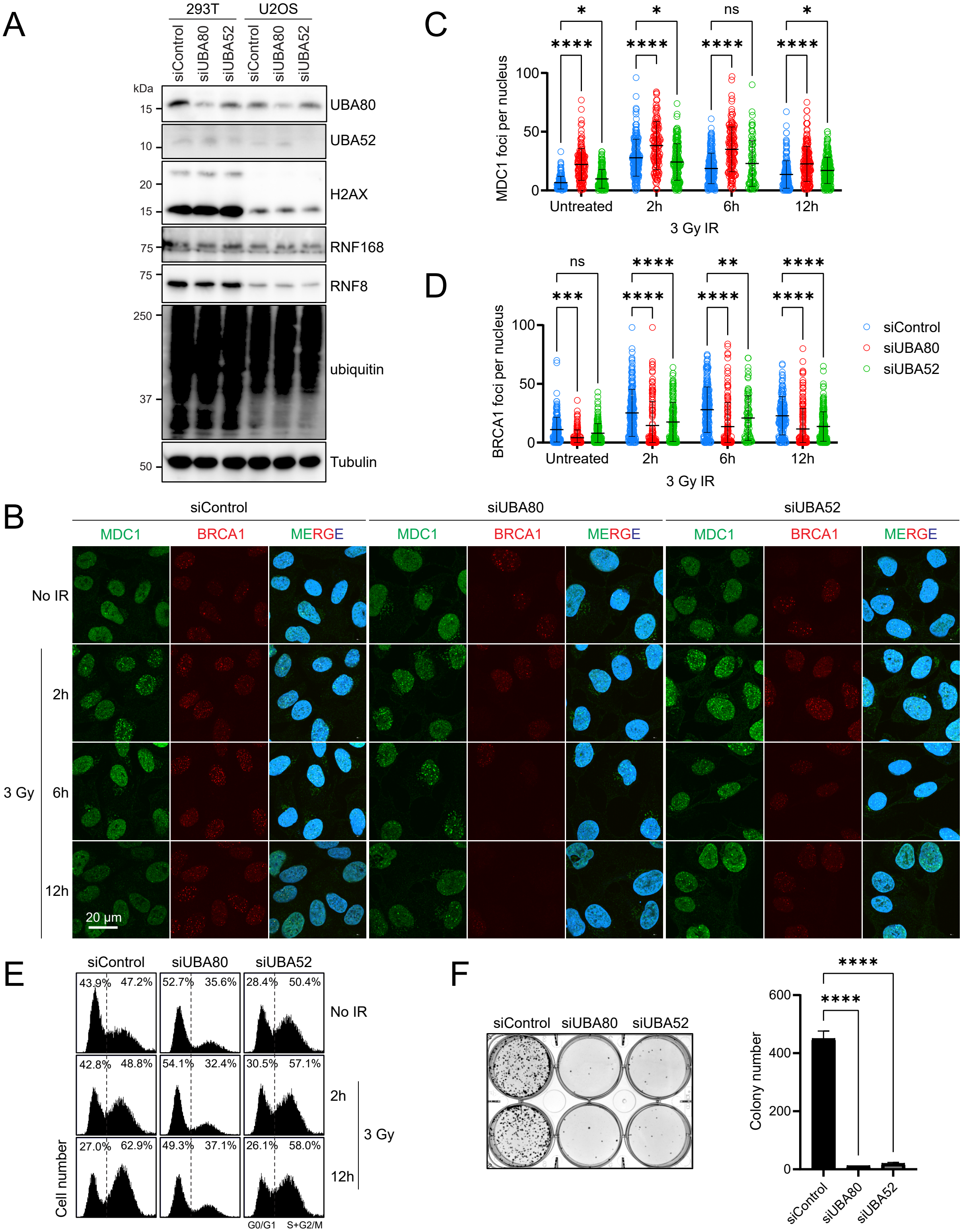
A
293T
U2OS
siControl
siUBA80
siUBA52
kDa
15
10
20
75
250
37
50
UBA80
UBA52
H2AX
RNF168
RNF8
ubiquitin
Tubulin
C
MDC1 foci per nucleus
100
50
0
Untreated
2h
6h
12h
3 Gy IR
ns
D
BRCA1 foci per nucleus
siControl
siUBA80
siUBA52
B
MDC1
BRCA1
MERGE
No IR
3 Gy
2h
6h
12h
20 μm
E
43.9% 47.2%
52.7% 35.6%
28.4% 50.4%
42.8% 48.8%
54.1% 32.4%
30.5% 57.1%
27.0% 62.9%
49.3% 37.1%
26.1% 58.0%
No IR
Cell number
G0/G1
S+G2/M
F
Colony number
600
400
200

Figure S4

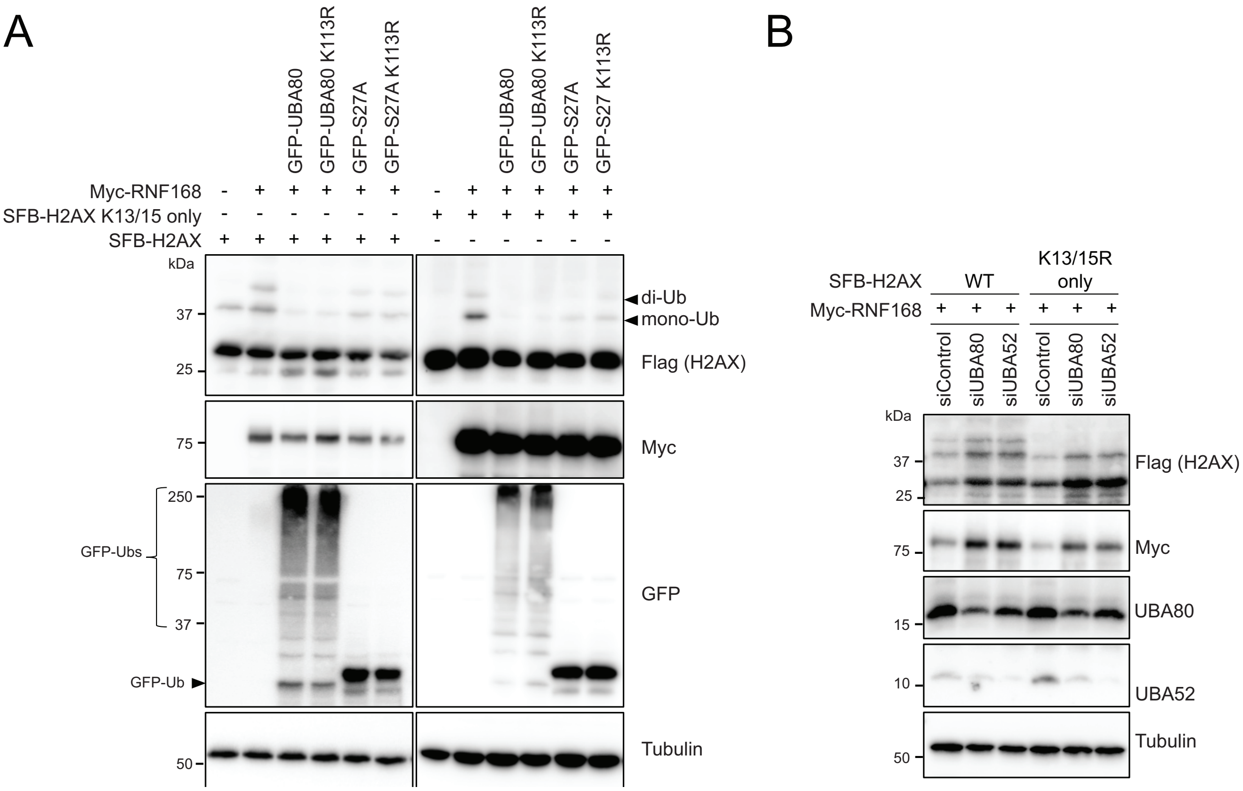
A
GFP-UBA80
GFP-UBA80 K113R
GFP-S27A
GFP-S27A K113R
GFP-UBA80
GFP-UBA80 K113R
GFP-S27A
GFP-S27 K113R
Myc-RNF168 - + + + + + - + + + + +
SFB-H2AX K13/15 only - - - - - - + + + + + +
SFB-H2AX + + + + + + - - - - - -
kDa
37
25
di-Ub
mono-Ub
Flag (H2AX)
75
Myc
GFP-Ubs
250
75
37
GFP-Ub
GFP
50
Tubulin
B
SFB-H2AX
WT
K13/15R only
Myc-RNF168 + + + + + +
siControl
siUBA80
siUBA52
siControl
siUBA80
siUBA52
kDa
37
25
Flag (H2AX)
75
Myc
15
UBA80
10
UBA52
50
Tubulin

Figure S5

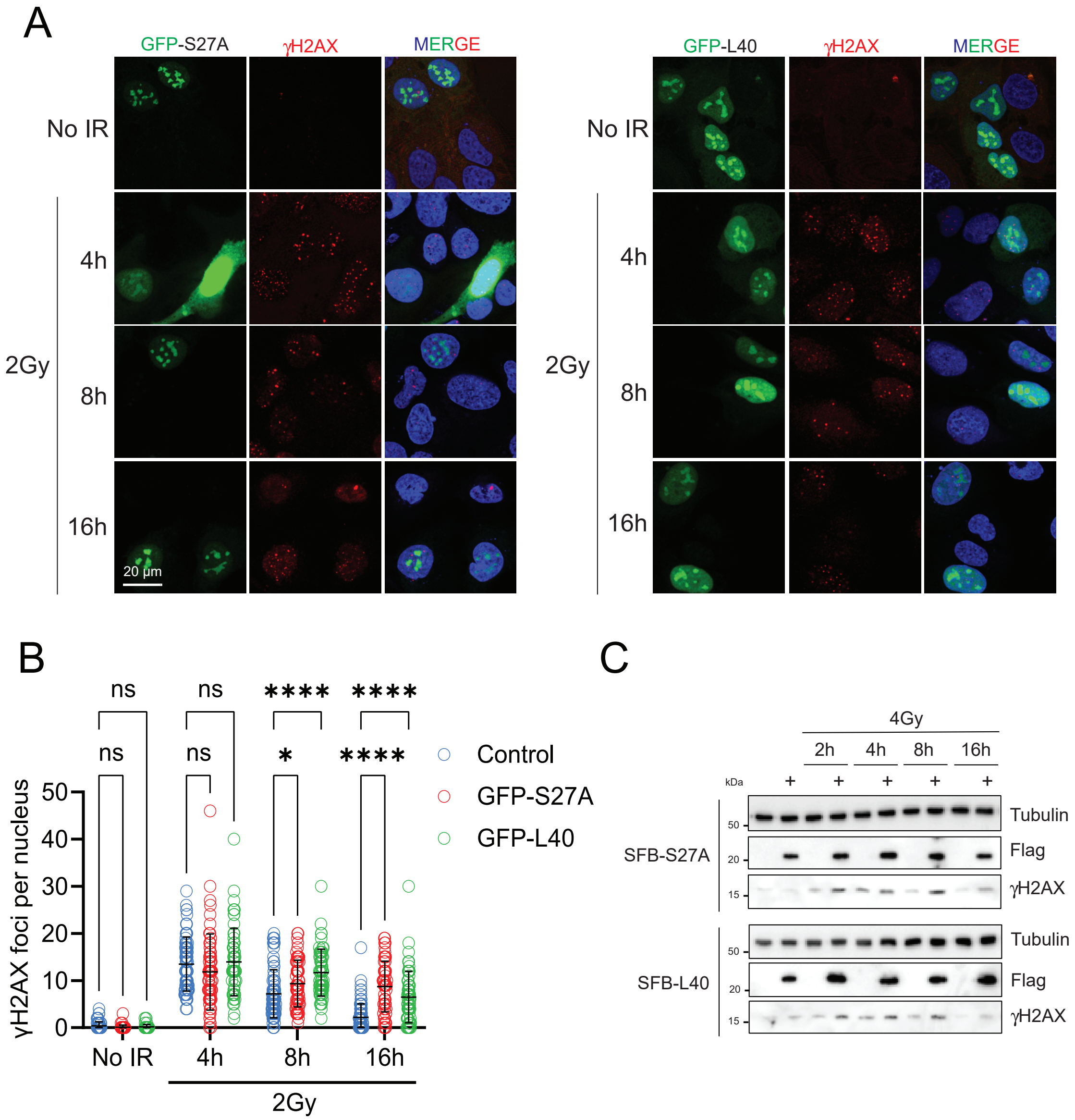
A
GFP-S27A
γH2AX
MERGE
GFP-L40
γH2AX
MERGE
No IR
2Gy
4h
8h
16h
20 μm
B
γH2AX foci per nucleus
ns
ns
****
****
*
****
Control
GFP-S27A
GFP-L40
No IR
4h
8h
16h
2Gy
C
4Gy
2h
4h
8h
16h
kDa
SFB-S27A
SFB-L40
Tubulin
Flag
γH2AX
50
20
15
